# Hexameric Aggregation Nucleation Core Sequences and Diversity of Pathogenic Tau Strains

**DOI:** 10.3390/pathogens11121559

**Published:** 2022-12-19

**Authors:** Ling Wu, Sidharth S. Madhavan, Christopher Tan, Bin Xu

**Affiliations:** 1 Biomanufacturing Research Institute and Technology Enterprise (BRITE), Department of Pharmaceutical Sciences, North Carolina Central University, Durham, NC 27707, USA; 2 Duke/UNC Alzheimer’s Disease Research Center, Durham, NC 27710, USA; 3 Department of Biochemistry, Virginia Polytechnic Institute and State University, Blacksburg, VA 24061, USA; 4 School of Neuroscience, Virginia Polytechnic Institute and State University, Blacksburg, VA 24061, USA

**Keywords:** tau aggregation, tauopathies, nucleation core sequences, amyloidogenicity, tau strains diversity

## Abstract

Tau aggregation associates with multiple neurodegenerative diseases including Alzheimer’s disease and rare tauopathies such as Pick’s disease, progressive supranuclear palsy, and corticobasal degeneration. The molecular and structural basis of tau aggregation and related diverse misfolded tau strains are not fully understood. To further understand tau-protein aggregation mechanisms, we performed systematic truncation mutagenesis and mapped key segments of tau proteins that contribute to tau aggregation, where it was determined that microtubule binding domains R2 and R3 play critical roles. We validated that R2- or R3-related hexameric PHF6 and PHF6* peptide sequences are necessary sequences that render tau amyloidogenicity. We also determined that the consensus VQI peptide sequence is not sufficient for amyloidogenicity. We further proposed single- and dual-nucleation core-based strain classifications based on recent cryo-EM structures. We analyzed the structural environment of the hexameric peptide sequences in diverse tau strains in tauopathies that, in part, explains why the VQI consensus core sequence is not sufficient to induce tau aggregation. Our experimental work and complementary structural analysis highlighted the indispensible roles of the hexameric core sequences, and shed light on how the interaction environment of these core sequences contributes to diverse pathogenic tau-strains formation in various tauopathy brains.

## 1. Introduction

**Figure 1:**
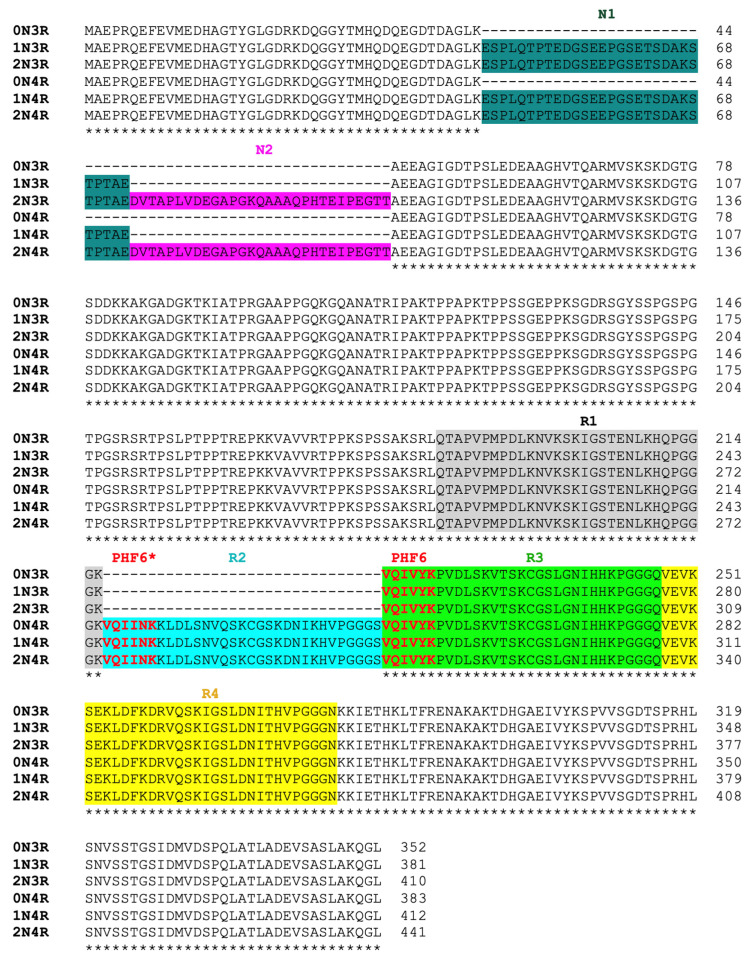
Human tau isoforms sequence alignment. Identical residues in the alignment are labelled with asterisks. Nucleation core sequences VQIVYK (PHF6) in all six isoforms and VQIINK (PHF6*) in 4R-tau isoforms are highlighted in red. N-terminal insert domain N1 is shaded in dark green, and N2 in purple. Microtubule repeat segments R1–R4 are highlighted in grey, cyan, green, and yellow, respectively.

Extracellular Aβ amyloid plaques and intraneuronal tau neurofibrillary tangles represent hallmark features of Alzheimer’s disease (AD) [1,2]. These abnormal structures were generally believed to cause the synaptic dysfunction and neuronal death that lead to the memory and cognitive deficits characteristic of AD patients [3,4]. Multiple lines of evidence have suggested that pathological changes in tangles correlate better with neuronal dysfunction than Aβ deposits [5,6]. Furthermore, a close relationship between tau aggregates and neuronal loss is well established in hippocampus and cerebral-cortex tissues [7]. Tau aggregates are present in multiple neurodegenerative diseases known as “tauopathies” [8,9], including AD, Pick’s disease (PiD), progressive supranuclear palsy (PSP), and corticobasal degeneration (CBD). Six tau isoforms are expressed in human brains. These isoforms contain either three or four microtubule-binding repeats (3R or 4R tau) and 0–2 N-terminal inserts (0N, 1N, or 2N tau) (Figure 1) [10,11,12]. Based on the tau isoforms that constitute the abnormal filaments, tauopathies can be divided into three groups. In AD and chronic traumatic encephalopathy (CTE), both 3R and 4R isoforms make up the neuronal inclusions [13,14]; 3R tau is found in PiD, whereas 4R tau isoforms are present in the filaments of PSP, CBD, argyophilic grain disease (AGD), and globular glial tauopathy (GGT) [15,16,17]. Transmission of the AD pathology and related tauopathies is not fully understood, but is thought to be through “prion-like seeding” mechanisms that ultimately yield intercellular spreading of toxic tau aggregates [18,19,20,21,22,23,24,25].

Significantly, isoform composition and the morphology of tau filaments can differ between tauopathies, suggesting the existence of distinct misfolded tau strains, molecular heterogeneity and complexity of these tauopathy diseases [26,27,28,29]. Recently, cryo-EM structures of tau filaments extracted from postmortem brains of different tauopathies patients have been solved. The diversity of the structural folds, which are in part reflected in the different morphology of tau filaments, allows us to gain insights into distinct misfolded tau strains. Furthermore, past experimental studies have recognized the important roles the microtubule-binding domain repeats (R1-R4) play in tau aggregation (Figure 1; [30]). Specifically, two nucleation core sequences in the microtubule binding region (MTBR), ^275^VQIINK^280^ (PHF6*) and ^306^VQIVYK^311^ (PHF6) were identified, each located at the beginning of R2 or R3 repeat region, respectively (Figure 1; [31]).

The objectives of this study are to understand sequence-specific human tau aggregation mechanisms and how key sequences contribute to the structural diversity of different tauopathies. Through systematic truncation mutagenesis, we not only mapped out R2 and R3 as key segments contributing to tau aggregation, but also validated that R2- and R3-related hexameric PHF6 and PHF6* peptide sequences are sufficient sequences that render tau amyloidogenicity, yet the consensus VQI peptide sequence alone is not sufficient. Furthermore, by analysing a collection of tau filament structures from diverse pathogenic tau strains, we proposed single- and dual-nucleation core-based strain classifications with respect to the hexameric PHF6 and PHF6* core sequences. Information on the structural environment of the hexameric peptide nucleation core sequences from a diverse set of pathogenic tau strains gives insights into why VQI consensus sequence alone is not sufficient for tau aggregation. Overall, our work not only highlighted the indispensable roles of hexameric core sequences, but also shed light on how their interaction environment contributes to diverse pathogenic tau strain formation, which is important for distinct tau strain propagation.

## 2. Materials and Methods

### 2.1. Plasmid Constructs

Expression vectors for his-tagged versions of all six wild-type human tau isoforms were kindly provided by the late Dr. Lester “Skip” Binder and Dr. Nicolas Kanaan of Michigan State University (via Prof. George Bloom of the University of Virginia). All the truncation constructs were first PCR-amplified of each specified segment of sequence from 2N4R tau plasmid (except for construct “C7”, where 2N3R tau plasmid was used as parent) and cloned into the same expression vector using Nde I and Xho I restriction sites. Oligo primers and related annealing temperatures for all the truncation mutagenesis are described in Supplemental Table S1. The PCR mixture preparation and thermocycling followed standard protocol. The PCR mixture consisted of 1 μL of 10X reaction buffer, 0.2 μL of 10 mM dNTP mix (Invitrogen, Waltham, MA, USA; cat. #18427013; 200 μM each final), 1 μL each of forward and reverse primers (200 nM each primer final), 0.2 μL of Pfu DNA polymerase (Agilent, Santa Clara, CA, USA; cat. #99903-844; 1 U/μL), and 10 ng of template in a total volume of 10 μL. PCR was performed with the following cycling profile: initial denaturation at 95 °C for 2 min, followed by 30 cycles of 30 sec denaturation at 95 °C, annealing temperature (different in primer pairs relevant to their Tm) for 30 sec, and extension at 72 °C for 1 min. The time for the final extension step was increased to 10 min. The PCR product was cleaned up by Qiagen PCR purification kit (cat. #28104), and then digested with NdeI/XhoI (Thermo Fisher Scientific, Waltham, MA, USA; cat. #ER0585 and ER0695), the fragments were then purified using the QIA quick gel extraction kit (Qiagen, Hilden, Germany; cat. #20021). The enzyme-digested product was ligated into the vector derived from NdeI/XhoI digested tau 2N4R product. Plasmid ligation was achieved by T4 DNA ligase (New England BioLabs, Ipswich, MA, USA; cat. #M0202S) at 22 °C overnight. The recombinant plasmids were transformed into E. coli XL1Blue (Agilent; cat. #200249) for amplification. Plasmid DNA from the resulting clones was purified using the QIAprep spin miniprep kit (Qiagen; cat. #27104). All constructs were designed with a his_6_-tag at their carboxy-termini to facilitate protein purification and were verified by DNA sequencing.

### 2.2. Recombinant Truncated Tau Fragments Expression and Purification

Recombinant tau protein expression and purification, in general, follows protocol as described [32,33]. Plasmids encoding human tau isoforms or truncated tau fragments were transformed into BL21-DE3 E. coli cells (Thermo Fisher Scientific; cat. #C600003). Overnight starter cultures of BL21-DE3 E. coli cells transformed with recombinant tau plasmids were inoculated into multi-liter LB broth at 1:50 dilution and 100 μg/mL ampicillin. Cultures were incubated at 37 °C, and shaken until OD_600_ reached between 0.5–0.6. Tau expression was induced using 1 mM IPTG and the cultures continued for an additional 4 h. BL21-DE3 cells containing expressed tau were pelleted and resuspended in 50 mM NaH_2_PO_4_, pH 8.0 and 300 mM NaCl (sonication lysis buffer) at a concentration of 20 mL/L of culture preparation and sonicated at 60% power in ten 30-s intervals over 10 min. Cell lysates were centrifuged and supernatant containing the protein was applied to Ni-NTA (Qiagen; cat. #30250) column equilibrated with sonication lysis buffer. The columns were washed with 40–60 times of bed volumes of column buffer followed by washing buffer (50 mM NaH_2_PO_4_, pH 8, 300 mM NaCl, and 20 mM imidazole). Recombinant tau proteins were then eluted using elution buffer (50 mM NaH_2_PO_4_, pH 8, 300 mM NaCl, and 200 mM imidazole). Fractions were tested for protein concentration using 5 μL of protein sample mixed with 10 μL Coomassie Protein Assay reagent (Thermo Fisher Scientific; cat. #23200). Pooled fractions were concentrated to 4 mL using 10 kD or 3 kD molecular weight cut-off spin columns (MilliporeSigma, Burlington, MA, USA; cat. #UFC901096 and 900324) and filtered using 0.22 μm low-binding Durapore PVDF membrane filters (MilliporeSigma; cat. #UFC40GV00 and UFC30GV00). Truncated tau proteins were further purified by FPLC using size-exclusion Superdex75 10/300 GL column (GE Healthcare, Chicago, IL, USA; cat. #17517401) in 1X PNE buffer (25 mM PIPES, 150 mM NaCl and 1 mM EDTA at pH 7.0). Purified tau fragments were evaluated by SDS-PAGE for purity and quantified by Pierce BCA protein assays (Thermo Fisher Scientific; cat. #23225).

### 2.3. Thioflavin-T Fluorescence Aggregation Kinetic Analysis

Fluorescence-based experiments were performed using a SpectraMax M5 plate reader (Molecular Devices, Sunnyvale, CA, USA). All kinetic reads were taken at 37 °C in non-binding all-black clear-bottom Greiner 96-well plates covered with optically clear films and stirred for 10 s prior to each reading. ThT fluorescence was measured at 444 nm and 491 nm as excitation and emission wavelengths. Each kinetic assay consisted of final concentrations of 30 μM tau protein, 60 μg/mL heparin, and 10 μM ThT [34]. Kinetic signals were collected until fluorescence signals plateaued, typically in 40–80 h.

### 2.4. Protein Sequence Alignment

Tau protein isoform alignment was performed by MAFFT version 7, a similarity-based multiple sequence alignment (MSA) program [35]. MSA program assumes all the input sequences are homologous and descended from a common ancestor. Fully conserved regions were labelled as asterisks.

### 2.5. Structural Analysis of Tau Strains from Different Tauopathies

Protein structural coordinates were retrieved from PDB Protein Data Bank (www.rcsb.org, accessed on 24 September 2022). For all tau cryo-EM structures, PDB access codes are 5O3L for the Alzheimer’s fold [36], 6NWP for the CTE fold [37], 6GX5 for the Pick’s fold [38], 6TJO for the CBD fold [39], 7P6D for the AGD fold [17], 7P65 for the PSP fold [17], 7P66 for the GGT fold [17], and 7P6A for the GPT fold [17]. PyMol molecular visualization system (Schrodinger, Inc., New York, NY, USA) was used for all protein structure display and atomic structural analysis applications. Hydrogen bonding length was limited to 3.3 angstrom in maximum and hydrophobic interaction distance cutoff was set for 5.0 angstrom in maximum.

## 3. Results

### 3.1. Truncation Mutagenesis of N-terminal & C-terminal Tau Sequences

**Figure 2:**
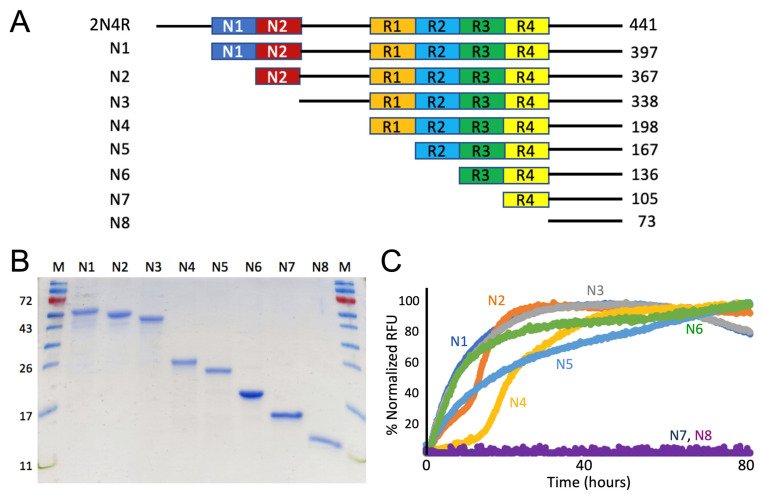
Mapping the key segments in 2N4R tau for protein aggregation using N-terminal truncation mutagenesis: critical role of the R3 repeat for tau aggregation. (**A**) N-terminal truncation constructs are schematically illustrated. All truncation constructs were cloned using 2N4R tau (total 441 residues) as the template. A his_6_-tag was added to each construct in the carboxy-terminal (not shown) to facilitate protein purification. Sizes for each construct in amino-acid residues are specified. (**B**) SDS-PAGE of purified recombinant N-terminal truncation tau proteins corresponding constructs schematically drawn in the panel A. Molecular weight markers are shown on either side and corresponding molecular weights (in kD) are labelled to the left of the gel. (**C**) ThT fluorescence assays for each N-terminal truncation mutant. A total of 15–30 μM of tau mutant proteins were incubated in 20 mM Tris pH 7.4 with 0.06 mg/mL of heparin at 37 °C for specified time periods. The results are representative of at least triplicated experiments.

**Figure 3:**
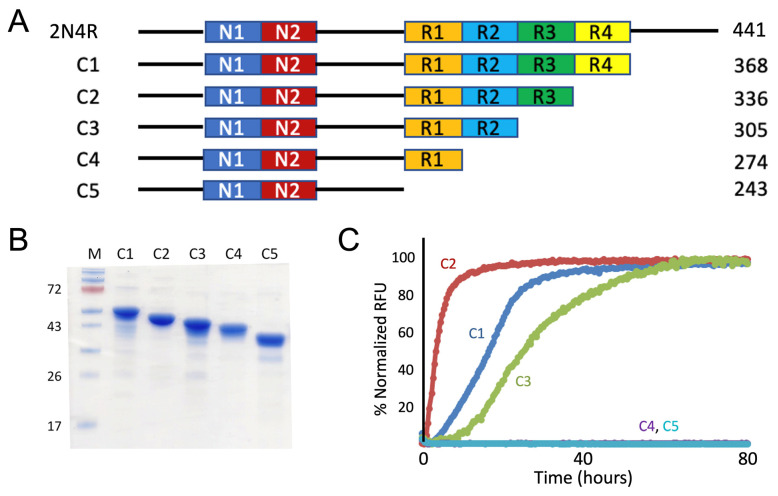
Mapping the key segments in 2N4R tau for protein aggregation using C-terminal truncation mutagenesis: critical role of the R2 repeat in tau aggregation. (**A**) Each C-terminal construct is schematically illustrated. All constructs were cloned using 2N4R tau (total 441 residues) as the template. A his_6_-tag was added to each construct in the carboxy terminal (not shown) to facilitate protein purification. Sizes for each construct in amino-acid residues are specified. (**B**) SDS-PAGE of purified recombinant C-terminal truncation tau proteins corresponding constructs schematically drawn in the panel A. Molecular weight markers (in kD) are labelled to the left of the gel. (**C**) ThT fluorescence assays for each truncation mutant. A total of 15–30 μM of tau mutant proteins were incubated in 20 mM Tris pH 7.4 with 0.06 mg/mL of heparin at 37 °C for specified time periods. The results are representative of at least triplicated experiments.

Using 2N4R tau full-length expression plasmid as a parent template, we performed PCR amplification reactions with appropriate oligo primer sequences (Table S1) and generated eight tau protein constructs truncated from the full-length sequence systematically deleted from the N-terminus (named as N1–N8; Figure 2A). Domain boundaries were set by distinct domains, such as N1, N2, proline-rich domain, R1, R2, R3, and R4. Similarly, we also generated five tau-protein sequences truncated from the carboxy-terminus (named as C1–C5; Figure 3A). A his_6_-tag was appended to either the N-terminus or C-terminus to facilitate protein purification. All the plasmids were sequence-verified to confirm there were no PCR-induced errors. Truncation mutants were transformed into expression host BL21DE3 bacteria. Ni-NTA affinity chromatography and size-exclusion chromatography were used to purify all the truncation mutants. Individual mutants were purified to homogeneity (>90% pure; Figure 2B and Figure 3B).

Protein-aggregation properties were characterized by standard ThT fluorescence- based assays for detection and monitoring the aggregation of the resulting truncated tau proteins for an extended period of time (40–80 h) until the fluorescence intensities were well in the plateau phases (Figure 2C and Figure 3C; [33,34]). For the N-terminal truncation mutants, it is evident that mutants N7 and N8 lost amyloidogenic properties while N1-N6 were competent in forming aggregation, as shown in Figure 2C. Based on the sequence difference boundary between N6 and N7, our ThT fluorescence results indicated the critical role of R3 repeat for aggregation. Similarly, for the C-terminal truncation mutants, mutants C4 and C5 lost amyloidogenicity (Figure 3C). Based on the sequence difference boundary between C3 and C4, ThT fluorescence data from the C-terminal truncation mutant set indicated the critical role of R2 repeat for aggregation. Overall, our systematic truncation mutagenesis work pointed to the critical roles R2 and R3 play in tau aggregation.

### 3.2. Recombinant Constructs with PHF6*, PHF6, and VQI Only Sequences

Based on our systematic truncation mutagenesis results and identification of R2 and R3 segments as two key segments in tau aggregation, and recognizing hexameric VQIINK (PHF6*) and VQIVYK (PHF6) as nucleation core sequences from past studies [31], we engineered three additional tau mutants C6–C8. All three constructs used C4, a non-amyloidogenic construct, as a carrier (Figure 3A): each was extended by VQIINK (C6), VQIVYK (C7), or the consensus sequence of PHF6 and PHF6*, VQI (C8). These constructs allowed us to test the hypothesis if the entire length of the hexameric sequences for both nucleation cores are necessary or they can be further shortened to consensus sequence VQI.

**Figure 4:**
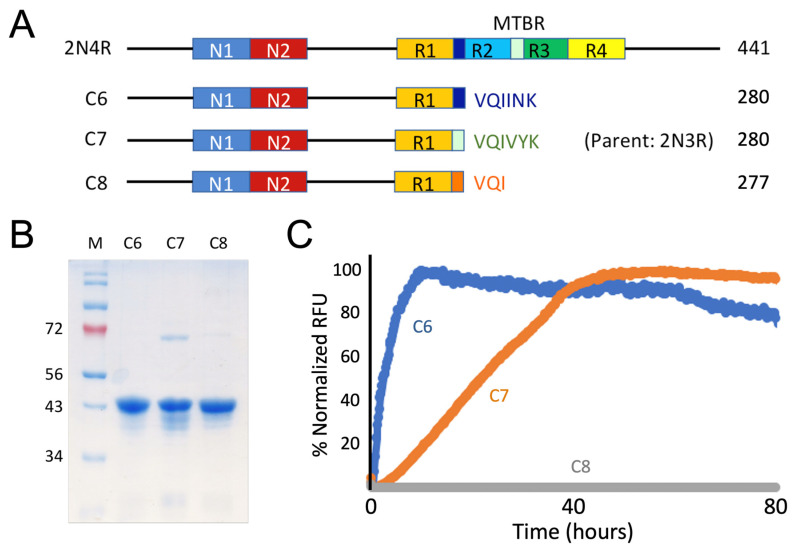
Mapping the key segments in 2N4R tau for aggregation using truncation mutagenesis: hexameric sequences PHF6 or PHF6* each is sufficient to be amyloidogenic but consensus VQI sequence is not. (**A**) Each construct is schematically illustrated. MTBR stands for microtubule binding region. Except for C7 construct, which was cloned using 2N3R tau as the template, all other constructs were cloned using 2N4R tau as the template. Hexameric core sequences VQIINK, VQIVYK, and their consensus sequence VQI are shown color-coded in blue, light green and orange, respectively. A his_6_-tag was added to each construct in the carboxy terminal (not shown) to facilitate protein purification. Amino-acid residue numbers for each construct are specified. (**B**) SDS-PAGE of purified recombinant C-terminal truncation mutants corresponding to constructs schematically drawn in the panel A. Molecular weight markers (in kD) are labelled to the left of the gel. (**C**) ThT fluorescence assays for each truncation mutant. A total of 15–30 μM of tau mutant proteins were incubated in 20 mM Tris pH 7.4 with 0.06 mg/mL of heparin at 37 °C for specified time periods. The results are representative of at least triplicated experiments.

Similarly to the truncation mutants described in 3.1., recombinant constructs C6–C8 were cloned using PCR amplification steps and appropriate oligos. Both C6 and C8 used 2N4R as parent template since the VQIINK sequence, in the case of C6 (or VQI sequence in the case of C8), from the R2 segment directly joins R1. As, in the 2N3R tau parent construct, VQIVYK sequence from R3 segment directly joins R1, the C7 construct was engineered using 2N3R as the parent template (Figure 4A). Recombinant C6, C7, and C8 were purified to homogeneity (>90% purity) after Ni-NTA affinity chromatography and size-exclusion chromatography (Figure 4B).

Protein aggregation was characterized by ThT fluorescence assays. As expected, C6 and C7 constructs demonstrated competency in forming tau amyloid (Figure 4C). However, the C8 construct (with VQI consensus sequence only) was not capable of forming tau amyloid. Comparison data of C6–C8 constructs showed that VQIINK or VQIVYK are alone sufficient to be amyloidogenic but shortened trimeric VQI sequence is not.

### 3.3. Hexameric Core Sequences in Diverse Pathogenic Tau Strains

**Figure 5:**
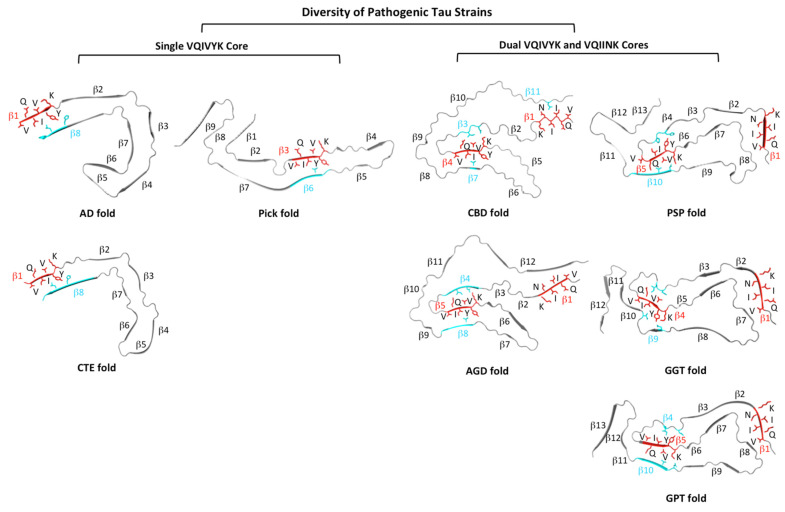
Molecular structure illustration of the diversity of pathogenic tau strains. Cryo-EM structures of tau protein folds in diverse tauopathies classified into single VQIVYK core strains (AD, CTE, and Pick folds) and dual VQIVYK and VQIINK core strains (CBD, AGD, PSP, GGT, and GPT folds). PDB access codes are 5O3L for AD fold, 6NWP for CTE fold, 6GX5 for Pick fold, 6TJO for CBD fold, 7P6D for AGD fold, 7P65 for PSP fold, 7P66 for GGT fold, and 7P6A for GPT fold. Hexameric core sequences are shown in red and their interaction amino acids/strands are shown in cyan. All β strands are labelled.

**Figure 6:**
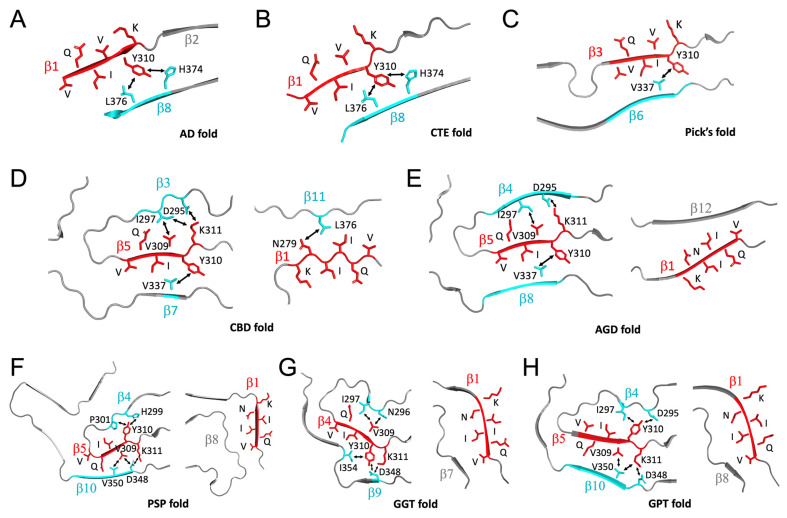
Atomic interactions involving residues **VYK** of VQIVYK (PHF6) or **INK** of VQIINK (PHF6*) hexameric core sequences in diverse tau strains. (**A**–**C**) Atomic interactions with VYK of PHF6 core sequence. (**D**–**H**) Atomic interactions with VYK of PHF6 sequence (left panels) or with INK of PHF6* sequence (right panels). Residue interactions are highlighted by arrows. Detailed inter-atomic interaction and distances are listed in Table S2. Hexameric core sequences are shown in red and their interacting amino acids/strands are shown in cyan. Relevant amino acids and β strands are labelled.

Cryo-EM structures of tau filaments extracted from diverse tauopathy brains with definitive neuropathological diagnosis have been solved recently [17,36,37,38,39]. The diversity of tau-protein structural folds from different tauopathies in part reflects the diversity of pathogenic tau strains. Tauopathies are often classified based on tau-isoform composition in the respective filaments. Based on the presence of the PHF6 and PHF6* nucleation core sequences, we propose tau structural folds in two classes (Figure 5): (1) Single VQIVYK Core. Three tauopathies, AD, CTE, and PiD each have a single VQIVYK core. (2) Dual VQIVYK and VQIINK Cores. CBD AGD, PSP, GGT, and GPT have both VQIVYK and VQIINK cores. In each category, they may be further sub-classified based on structural folds. The CTE structural fold is quite similar to the “C”-shaped AD fold, but is significantly different from the elongated/extended Pick’s fold. In the dual nucleation core, CBD and AGD share similar structural folds; PSP fold, GGT fold, and GPT fold, however, demonstrated a different structural fold. Regarding two-nucleation-core hexameric sequences, VQIVYK is present in all tauopathy structures so far determined; however, VQIINK is only presented in tau filaments structures with the presence of dual-hexamer core sequences (CBD, AGD, PSP, GGT, and GPT). Comparing nucleation sequences in various tau structural folds and each core sequence’s interacting environment, we speculated that VQIVYK likely serves as the primary nucleation core because of its tight packing with other interacting elements. The VQIINK sequence likely serves as a secondary nucleation core since it has much fewer interactions with neighbouring strands or residues. The N-terminals of CBD, AGD, PSP, GGT, and GPT folds, where the VQIINK sequence is located, only loosely pack against the structural fold with few interactions with their neighbouring residues: very few intramolecular bonds (< 5.0 angstrom in distance) were found involving INK residues in the VQIINK core (Figure 5 and Figure 6 and Table S2).

### 3.4. Why Consensus VQI Sequence Is not Sufficient for Tau Aggregation?

We further investigated the atomic basis of why the consensus sequence VQI is not sufficient to induce tau aggregation while either VQIVYK or VQIINK sequence is sufficient. We analysed the atomic interactions changes (loss of interactions) due to the absence of “VYK” in VQIVYK core sequence or “INK” in VQIINK core sequence in all available structural folds of tau filaments (Figure 6 and Table S2). In the single-nucleation-core AD fold, CTE fold, and Pick’s fold (Figure 6A–C), intra-molecular interactions between Tyr310 of VQIVYK with spatial neighbours Leu376 and His374 (in the AD fold and CTE fold), or between Tyr310 with spatial neighbour Val337 (in the Pick’s fold) will be lost in the absence of “VYK” residues. In the structural folds with dual nucleation cores, intra-molecular bonds between Val309 and Ile297, Tyr310 and Val337, Lys311 and Ile297, Lys311 and Asp295 related to core VQIVYK, and Asn279 and Leu376 related to core VQIINK, will be lost in the absence of “VYK” or “INK” in CBD fold (Figure 6D); similarly, bonds between Val309 and Ile297, Tyr310 and Val337, and Lys311 and Asp295 relevant to core VQIVYK will be lost in AGD fold (Figure 6E). In PSP, GGT, and GPT structural folds with dual cores, intra-molecular bonds between Val309 and Val350, Val309 and Asp348, Tyr310 and Pro301, Tyr310 and His299, Lys311 and Asp348 in PSP fold (Figure 6F), bonds between Val309 and Asn296, Val309 and Ile297, Tyr310 and Asp348, Tyr310 and Ile354, Lys311 and Asp348 in GGT fold (Figure 6G), Val309 and Val350, Tyr310 and Ile297, Tyr310 and Asp295, Lys311 and Val350, and Lys311 and Asp348 in GPT fold (Figure 6H), will be lost. Such loss of multiple intra-molecular bonds may significantly destabilize each structural fold to a degree that the remaining VQI-induced intra-molecular bonds are not strong enough to form a nucleation core for amyloid growth.

The nature of interactions between “VYK” from VQIVYK and its neighbouring residues is primarily hydrophobic interactions supplemented with a few hydrogen bonds (Table S2). There are very few interactions between “INK” from VQIINK and its neighbours in the dual hexameric core tau structures (Figure 6). It is interesting to observe that VQIVYK nucleation core has more intra-molecular bonds with its neighbouring residues than those for VQIINK in the dual-core structural folds (Figure 6D–H), which is further supported by the evidence that VQIVYK is the only core present in the tau structural folds with a single nucleation core (Figure 6A–C). From the structural point of view, we propose VQIVYK may be the primary drive force for tau aggregation and VQIINK may play secondary roles in tau aggregation.

## 4. Discussion

We determined the critical roles of R2 and R3 in tau aggregation and further validated that R2- or R3-related hexameric PHF6 and PHF6* peptide sequences are necessary sequences that render tau amyloidogenicity. These results may have significant values for future translational applications. For example, we can use R2, R3, R2–R3, or PHF6, PHF6* peptides as surrogates for tau-aggregation mechanistic studies or inhibitor screening [40]. Significantly, R3 or R2–R3 each represents a key segment in determining 3R-tau or 4R-tau isoform aggregation; therefore, these two peptides may be used for 3R- or 4R-tau isoform-specific aggregation mechanistic studies or inhibitor screening.

While significant past work had been performed to elucidate major roles played by R1–R4 repeating segments in tau aggregation, the functions of various domains and segments of tau proteins are not fully understood. For examples, past studies identified the critical roles of VQIVYK and VQIINK hexameric nucleation core sequences [31]. In this study, we provided experimental data to demonstrate that entire hexameric sequences are necessary and VQI alone is not sufficient for tau amyloidogenicity. We further complemented experimental data with structure-analysis-based explanations using multiple cryo-EM atomic structures of tau strains extracted from different postmortem tauopathy brains. Significant questions remain regarding the roles of other segments of the tau protein, for example, what are the roles of N-terminal N1 and N2 insert domains in tau aggregation? Our recent study showed significant kinetic differences in tau aggregation from pair-wise comparison of tau isoforms, 0N3R/0N4R, 1N3R/1N4R, and 2N3R/2N4R, where the differences in the t_1/2_ fold of changes may be assigned to the presence or absence of the N1 and N2 segments of the isoforms evaluated [33,41]. Novel roles of N1 and N2 remain to be elucidated and this is an interesting future direction.

It appears that the VQIVYK (PHF6) core sequence may be the primary nucleation core for tau aggregation and VQIINK plays a secondary role. This is supported by the evidence that there are more intra-molecular interactions between PHF6 and its environment versus those for PHF6* (Figure 6D–H). However, inhibitors based on the structure of the VQIVYK segment only partially inhibit full-length tau aggregation and are ineffective at inhibiting seeding by full-length fibrils [42]. Designed VQIINK inhibitors, however, can more potently block seeding by full-length tau while VQIVYK-based inhibitors cannot. Therefore, it is still an open question as to which core sequence plays the dominant roles in driving tau aggregation and seeding. In physiological and pathological states, other factors may also play important roles in driving tau aggregation, as discussed below.

Multiple cell-based and in-vivo mice-based tau-seeding experiments provided strong evidence that tau protein has essential characteristics of a prion [19,20,27]. Prion-like propagation of tau aggregates may, therefore, underlie the disease pathogenesis and progression of neurodegenerative tauopathies. Recent groundbreaking cryo-EM structures of tau filaments from patients with AD, PiD, CBD, and other primary tauopathies further provided atomic evidence of different molecular conformers for a list of distinct neurodegenerative tauopathies [17,36,37,38,39]. Utilizing concepts and techniques such as RT-QuIC originally used in prion disease research, we and others demonstrated “prion-like” tau-isoform seeding activities in diseased AD and related tauopathy brains [33,43,44,45]. Tau proteins from in-vivo sources, including tauopathy brains, are heavily “decorated” by post-translational modifications (PTMs). Such modifications not only include well-recognized hyperphosphorylation, but also acetylation, ubiquitination, and methylation [46,47,48]. Therefore, it will be interesting and important to understand the roles of site-specific PTMs in tau aggregation, as these modifications exist in vivo and may play significant roles in tauopathy pathogenesis [47,49]. What are the roles of the six human tau isoforms in tau aggregation: do they play any role in disease progression in the context of rapid and slow progressive AD patients given that biochemical data suggests that they have different aggregation kinetics? How can we utilize the diversity of pathogenic tau strains and their atomic structures to design novel diagnostic tests for translation? New technological advances such as advanced mass spectrometry and ultrasensitive misfolded protein detection will certainly facilitate addressing many such important questions.

## Data Availability

The datasets used and/or analysed during the current study are available from the corresponding authors upon reasonable request.

## Supplementary Materials

The following supporting information can be downloaded at: https://www.mdpi.com/article/10.3390/pathogens11121559/s1, Table S1: Oligo Primer Sequence Information for Truncation Mutagenesis. PCR annealing temperatures are calculated by IDT OligoAnalyzer Tool based on primer pair sequences and experimental reaction conditions; Table S2: Atomic interactions involving residues VYK of VQIVYK (PHF6) or INK of VQIINK (PHF6*) hexameric core sequences in diverse tau strains. Corresponding intra-molecular interactions are highlighted by arrows in Figure 6. Inter-atomic distances are measured using Pymol Molecular Display Software (Version 2.5.4—Updated 17 August 2022).

## Abbreviations

AD: Alzheimer’s disease; AGD: Argyophilic grain disease; CBD: Corticobasal degeneration; CNS: central nervous system; Cryo-EM: cryo-electron microscopy; CTE: chronic traumatic encephalopathy; GGT: globular glial tauopathy; GPT: GGT/PSP hybrid tauopathy; MSA: multiple sequence alignment; MT: microtubule; NFT: neurofibrillary tangle; PBS: phosphate-buffered saline; PHF: paired helical filament; PiD: Pick’s disease; PSP: progressive supranuclear palsy; PTM: post-translational modification; RFU: relative fluorescence unit; ThT: thioflavin T.
