# Supplementary material for: Hexameric Aggregation Nucleation Core Sequences and Diversity of Pathogenic Tau Strains
Source: Pathogens. 2022 Dec 19;11(12):1559. doi: 10.3390/pathogens11121559 (PMC9784471; doi:10.3390/pathogens11121559)
Supplement: Supplementary file 1 [file pathogens-11-01559-s001.zip › pathogens-1965424-supplementary.pdf]

Supporting Information (SI)

Hexameric Aggregation Nucleation Core Sequences and Diversity of Pathogenic Tau Strains

Ling Wu [1,2], Sidharth S. Madhavan [3,4], Christopher Tan [3] and Bin Xu [1,2,3,4,*]

[1] Biomanufacturing Research Institute & Technology Enterprise (BRITE), Department of Pharmaceutical Sciences, North Carolina Central University, Durham, NC 27707, USA

[2] Affiliated Member, Duke/UNC Alzheimer's Disease Research Center, Durham, NC 27710, USA

[3] Departments of Biochemistry, Virginia Polytechnic Institute and State University, Blacksburg, VA 24061, USA

[4] School of Neuroscience, Virginia Polytechnic Institute and State University, Blacksburg, VA 24061, USA

Table S1. Oligo Primer Sequence Information for Truncation Mutagenesis. PCR annealing temperatures are calculated by IDT OligoAnalyzer Tool based on primer pair sequences and experimental reaction conditions.

Primers		Sequence (5'-3')	Annealing Temp (°C)
N1	Forward	CATTCATATGGAATCTCCCCTGCAG	65.0
	Reverse	TTAACTCGAGTCAATGATGATGATGATGATGCAAACCCTGCTTGGCCAGG	
N2	Forward	CATTCATATGGTGACAGCACCCTTA	64.7
	Reverse	TTAACTCGAGTCAATGATGATGATGATGATGCAAACCCTGCTTGGCCAGG	
N3	Forward	CATTCATATGGAAGAAGCAGGCATT	63.7
	Reverse	TTAACTCGAGTCAATGATGATGATGATGATGCAAACCCTGCTTGGCCAGG	
N4	Forward	AATTCATATGCAGACAGCCCCCGTGCCCAT	72.0
	Reverse	TTAACTCGAGTCAATGATGATGATGATGATGCAAACCCTGCTTGGCCAGG	
N5	Forward	TTAACATATGGTGCAGATAATTAATAAG	58.6
	Reverse	TTAACTCGAGTCAATGATGATGATGATGATGCAAACCCTGCTTGGCCAGG	
N6	Forward	TTAACATATGGTGCAAATAGTCTAC	59.1
	Reverse	TTAACTCGAGTCAATGATGATGATGATGATGCAAACCCTGCTTGGCCAGG	
N7	Forward	TTAACATATGGTGGAAGTAAAATC	57.1
	Reverse	TTAACTCGAGTCAATGATGATGATGATGATGCAAACCCTGCTTGGCCAGG	
N8	Forward	TTAACATATGAAAAAGATTGAAAC	54.5
	Reverse	TTAACTCGAGTCAATGATGATGATGATGATGCAAACCCTGCTTGGCCAGG	
C1	Forward	CATTCATATGGCTGAGCCCCGCCAG	71.0
	Reverse	TTAACTCGAGTCAATGATGATGATGATGATGATTTCCTCCGCCAGGGACGT	
C2	Forward	CATTCATATGGCTGAGCCCCGCCAG	71.0
	Reverse	TTAACTCGAGTCAATGATGATGATGATGATGCTGGCCACCTCCTGGTTTAT	
C3	Forward	CATTCATATGGCTGAGCCCCGCCAG	71.0
	Reverse	TTAACTCGAGTCAATGATGATGATGATGATGACTGCCGCCTCCCGGGACGT	
C4	Forward	CATTCATATGGCTGAGCCCCGCCAG	71.0
	Reverse	TTAACTCGAGTCAATGATGATGATGATGATGCTTCCCGCCTCCCGGCTGGT	
C5	Forward	CATTCATATGGCTGAGCCCCGCCAG	71.0
	Reverse	TTAACTCGAGTCAATGATGATGATGATGATGCAGGCGGCTCTTGGCGGAAG	
C6	Forward	CATTCATATGGCTGAGCCCCGCCAG	71.0
	Reverse	TTAACTCGAGTCAATGATGATGATGATGATGCTTATTAATTATCTGCACCTTC	
C7	Forward	CATTCATATGGCTGAGCCCCGCCAG	71.0
	Reverse	TTAACTCGAGTCAATGATGATGATGATGATGTTTGTAGACTATTTGCACCTTC	
C8	Forward	CATTCATATGGCTGAGCCCCGCCAG	71.0
	Reverse	TTAACTCGAGTCAATGATGATGATGATGATGTATCTGCACCTTCCCGCCTC	

Table S2. Atomic interactions involving residues VYK of VQIVYK (PHF6) or INK of VQIINK (PHF6*) hexameric core sequences in diverse tau strains. Corresponding intra-molecular interactions are highlighted by arrows in Figure 6. Inter-atomic distances are measured using Pymol Molecular Display Software.

Protein	Residue 1	Residue 2	Distance (Å)	Type of bonding
**Tau (Alzheimer's fold)**				
PDB access code:				
5O3L	Y310/CB	L376/CD2	4.8	Hydrophobic
	Y310/CG	L376/CD2	4.5	Hydrophobic
	Y310/CD1	L376/CD2	4.0	Hydrophobic
	Y310/CD1	L376/CG	5.0	Hydrophobic
	Y310/CD1	L376/CB	4.9	Hydrophobic
	Y310/CE1	L376/CB	4.6	Hydrophobic
	Y310/CZ	H374/CG	4.9	Hydrophobic
	Y310/CZ	H374/CD2	4.7	Hydrophobic
	Y310/CZ	H374/CB	4.7	Hydrophobic
**Tau (CTE fold)**				
PDB access code:				
6NWP	Y310/CB	L376/CD2	3.8	Hydrophobic
	Y310/CB	L376/CG	5.0	Hydrophobic
	Y310/CG	L376/CB	4.7	Hydrophobic
	Y310/CG	L376/CG	4.9	Hydrophobic
	Y310/CG	L376/CD2	4.1	Hydrophobic
	Y310/CD1	L376/CB	4.2	Hydrophobic
	Y310/CD1	L376/CG	4.6	Hydrophobic
	Y310/CD1	L376/CD1	4.8	Hydrophobic
	Y310/CD1	L376/CD2	4.1	Hydrophobic
	Y310/CD2	L376/CD2	4.9	Hydrophobic
	Y310/CD2	H374/CE1	4.9	Hydrophobic
	Y310/CE1	L376/CA	4.9	Hydrophobic
	Y310/CE1	L376/CB	4.3	Hydrophobic
	Y310/CE1	L376/CG	5.0	Hydrophobic
	Y310/CE2	H374/CB	4.2	Hydrophobic
	Y310/CE2	H374/CG	4.3	Hydrophobic
	Y310/CE2	H374/CE1	4.6	Hydrophobic
	Y310/CZ	H374/CA	4.7	Hydrophobic
	Y310/CZ	H374/CB	4.3	Hydrophobic
	Y310/CZ	H374/CG	4.5	Hydrophobic
	Y310/CZ	H374/CE1	4.8	Hydrophobic
	Y310/CZ	L376/CB	4.8	Hydrophobic
	Y310/OH	H374/ND1	3.0	Hydrogen bond
**Tau (Pick's fold)**				
PDB access code:				
6GX5	Y310/CD2	V337/CG2	4.4	Hydrophobic
	Y310/CE2	V337/CG2	4.8	Hydrophobic
**Tau (CBD fold)**				
PDB access code:				
6TJO	V309/CB	I297/CG1	4.7	Hydrophobic
	V309/CB	I297/CG2	4.9	Hydrophobic
	V309/CG1	I297/CG1	4.4	Hydrophobic
	V309/CG1	I297/CD1	4.7	Hydrophobic
	V309/CG2	I297/CB	4.8	Hydrophobic
	V309/CG2	I297/CG1	4.2	Hydrophobic
	V309/CG2	I297/CG2	3.9	Hydrophobic
	Y310/CD1	V337/CG2	4.7	Hydrophobic
	K311/CD	D295/CG	4.5	Hydrophobic
	K311/CD	I297/CD1	5.0	Hydrophobic
	K311/CE	D295/CG	4.1	Hydrophobic
	K311/CE	I297/CD1	4.6	Hydrophobic
	N279/CB	L376/CD1	4.4	Hydrophobic
**Tau (AGD fold)**				
PDB access code:				
7P6D	V309/CB	I297/CG1	4.4	Hydrophobic
	V309/CG1	I297/CD1	4.3	Hydrophobic
	Y309/CG2	I297/CB	4.5	Hydrophobic
	V309/CG2	I297/CG2	4.5	Hydrophobic
	Y309/CG2	I297/CD1	3.8	Hydrophobic
	Y310/CD1	V337/CG2	4.3	Hydrophobic
	Y310/CE1	V337/CG2	4.4	Hydrophobic
	K311/CD	D295/CG	4.5	Hydrophobic
	K311/CE	D295/CG	4.2	Hydrophobic
	K311/NZ	D295/OD2	2.9	Hydrogen bond
	K311/NZ	D295/OD1	3.0	Hydrogen bond
**Tau (PSP fold)**				
PDB access code:				
7P65	V309/CB	D348/CG	5.0	Hydrophobic
	V309/CB	V350/CG2	4.8	Hydrophobic
**Tau (PSP fold)**				
PDB access code:				
7P65	Y310/CB	P301/CG	4.7	Hydrophobic
Continued	Y310/CG	P301/CB	4.3	Hydrophobic
	Y310/CG	P301/CG	4.1	Hydrophobic
	Y310/CD1	P301/CG	4.5	Hydrophobic
	Y310/CD2	P301/CA	4.9	Hydrophobic
	Y310/CD2	P301/CB	3.6	Hydrophobic
	Y310/CD2	P301/CG	3.8	Hydrophobic
	Y310/CD2	P301/CD	4.6	Hydrophobic
	Y310/CE1	H299/CB	4.3	Hydrophobic
	Y310/CE1	H299/CG	4.3	Hydrophobic
	Y310/CE1	H299/CE1	4.6	Hydrophobic
	Y310/CE1	P301/CG	4.7	Hydrophobic
	Y310/CE1	P301/CD	5.0	Hydrophobic
	Y310/CE2	P301/CA	4.9	Hydrophobic
	Y310/CE2	P301/CB	3.9	Hydrophobic
	Y310/CE2	P301/CG	3.9	Hydrophobic
	Y310/CE2	P301/CD	4.3	Hydrophobic
	Y310/CZ	H299/CA	4.7	Hydrophobic
	Y310/CZ	H299/CB	4.0	Hydrophobic
	Y310/CZ	H299/CG	4.2	Hydrophobic
	Y310/CZ	H299/CE1	4.6	Hydrophobic
	Y310/CZ	P301/CB	4.7	Hydrophobic
	Y310/CZ	P301/CG	4.4	Hydrophobic
	Y310/CZ	P301/CD	4.5	Hydrophobic
	Y310/OH	H299/ND1	2.5	Hydrogen bond
	K311/CE	D348/CG	3.8	Hydrophobic
	K311/NZ	D348/OD2	2.9	Hydrogen bond
**Tau (GGT fold)**				
PDB access code:				
7P66	V309/CB	N296/CA	5.0	Hydrophobic
	V309/CB	N296/CB	4.3	Hydrophobic
	V309/CB	N296/CG	4.5	Hydrophobic
	V309/CG1	N296/CA	4.9	Hydrophobic
	V309/CG1	N296/CB	4.2	Hydrophobic
	V309/CG1	N296/CG	4.1	Hydrophobic
	V309/CG2	N296/CA	4.3	Hydrophobic
	V309/CG2	N296/CB	3.9	Hydrophobic
	V309/CG2	N296/CG	4.6	Hydrophobic
	V309/CG2	I297/CG1	3.9	Hydrophobic
	V309/CG2	I297/CD1	4.2	Hydrophobic
	Y310/CD1	I354/CG2	5.0	Hydrophobic
	Y310/CD1	I354/CD1	5.0	Hydrophobic
	Y310/CE1	D348/CG	5.0	Hydrophobic
	Y310/CE1	I354/CD1	4.7	Hydrophobic
	Y310/CE2	D348/CB	4.6	Hydrophobic
	Y310/CE2	D348/CG	4.0	Hydrophobic
	Y310/CZ	D348/CG	3.9	Hydrophobic
	Y310/OH	D348/OD1	2.8	Hydrogen bond
	Y310/OH	D348/OD2	3.1	Hydrogen bond
	K311/CE	D348/CB	4.8	Hydrophobic
**Tau (GPT fold)**				
PDB access code:				
7P6A	V309/CB	V350/CG1	4.9	Hydrophobic
	V309/CG1	V350/CB	4.7	Hydrophobic
	V309/CG1	V350/CG1	4.3	Hydrophobic
	V309/CG1	V350/CG2	5.0	Hydrophobic
	V309/CG2	V350/CG1	4.2	Hydrophobic
	Y310/CG	I297/CD1	4.9	Hydrophobic
	Y310/CD2	I297/CG1	4.4	Hydrophobic
	Y310/CD2	I297/CD1	3.6	Hydrophobic
	Y310/CE2	D295/CB	5.0	Hydrophobic
	Y310/CE2	I297/CB	4.9	Hydrophobic
	Y310/CE2	I297/CG1	4.0	Hydrophobic
	Y310/CE2	I297/CD1	3.7	Hydrophobic
	Y310/CZ	D295/CB	4.4	Hydrophobic
	Y310/CZ	I297/CD1	5.0	Hydrophobic
	K311/CD	D348/CB	5.0	Hydrophobic

V309/CG1	D348/CB	4.9	Hydrophobic	K311/CD	V350/CG2	4.5	Hydrophobic
V309/CG1	D348/CG	4.0	Hydrophobic	K311/CE	D348/CB	3.9	Hydrophobic
V309/CG2	V350/CB	4.9	Hydrophobic	K311/CE	D348/CG	4.0	Hydrophobic
V309/CG2	V350/CG1	4.8	Hydrophobic	K311/CE	V350/CG2	4.7	Hydrophobic
V309/CG2	V350/CG2	3.8	Hydrophobic	K311/NZ	D348/OD2	2.4	Hydrogen bond
Y310/CB	P301/CB	4.8	Hydrophobic				
